# Assessing insulin resistance: the triglyceride-glucose index as a predictor of survival in nasopharyngeal carcinoma

**DOI:** 10.3389/fphys.2025.1716333

**Published:** 2026-01-05

**Authors:** Xin Hua, Fei Xu, Xu-Xin Lin, Yong-Miao Lin, Zhi-Qing Long, Si-Fen Wang, Fang-Fang Duan, Chao Zhang, Xin Huang, Wen Xia, Wen-Chao Li, Ao-Qiang Chen, De-Huan Xie, Sha-Sha Du

**Affiliations:** 1 Department of Radiation Oncology, Guangdong Provincial People’s Hospital (Guangdong Academy of Medical Sciences), Southern Medical University, Guangzhou, China; 2 Department of Radiation Oncology, Shanghai Jiao Tong University Medical School Affiliated Ruijin Hospital, Shanghai, China; 3 School of Medicine South China University of Technology, Guangzhou, China; 4 Sun Yat-sen University Cancer Center, State Key Laboratory of Oncology in South China, Guangdong Provincial Clinical Research Center for Cancer, Guangdong Key Laboratory of Nasopharyngeal Carcinoma Diagnosis and Therapy, Guangzhou, China; 5 Department of Oncology, The Sixth Affiliated Hospital, School of Medicine, South China University of Technology, Foshan, China

**Keywords:** triglyceride-glucose index, nasopharyngeal carcinoma, concurrent chemoradiotherapy, prognosis, nomogram

## Abstract

**Background:**

The triglyceride-glucose (TyG) index, a simple marker of insulin resistance, has shown prognostic value in various malignancies. However, its predictive utility for survival in nasopharyngeal carcinoma (NPC) patients remains largely unexamined. This study aimed to assess the prognostic value of the TyG index and to develop novel predictive models for survival outcomes in NPC.

**Methods:**

We retrospectively analyzed 833 NPC patients treated with concurrent chemoradiotherapy (CCRT). All patients were staged according to the 8th edition of the American Joint Committee on Cancer (AJCC)/Union for International Cancer Control (UICC) TNM staging system. The TyG index was calculated as ln (fasting triglycerides × fasting glucose). Primary and secondary endpoints were overall survival (OS), locoregional recurrence-free survival (LRFS), and distant metastasis-free survival (DMFS), respectively. We utilized univariate and multivariate Cox proportional hazards models to identify independent prognostic factors and subsequently constructed and validated nomograms.

**Results:**

A low TyG index was significantly associated with better survival outcomes, serving as an independent predictor for OS (hazard ratio [HR] = 0.534; P = 0.007), LRFS (HR = 0.423; P < 0.001), and DMFS (HR = 0.575; P = 0.010) in multivariate analysis. The newly developed nomograms demonstrated favorable discriminative performance, significantly outperforming the conventional TNM staging system (concordance index [C-index] for OS: 0.722 vs. 0.634).

**Conclusion:**

The TyG index is a readily available, powerful prognostic biomarker for NPC patients. Incorporating the TyG index into prognostic nomograms offers a superior tool for individualized risk stratification and treatment planning, representing a valuable advancement over traditional staging systems.

## 1 Introduction

Nasopharyngeal carcinoma (NPC) exhibits distinct epidemiological patterns and clinical characteristics that distinguish it from other head and neck malignancies (Chen et al., 2019). While contemporary treatment strategies incorporating intensity-modulated radiotherapy combined with platinum-based concurrent chemoradiotherapy have significantly improved patient outcomes, substantial prognostic heterogeneity remains among patients with identical tumor-node-metastasis classifications (Wong et al., 2021). This variability underscores the critical need for novel biomarkers that reflect host metabolic status and enable refined personalized risk stratification.

Accumulating evidence suggests that metabolic disturbances play pivotal roles in NPC development and progression. Epidemiological studies from endemic regions have documented significant associations between type 2 diabetes mellitus and increased head and neck cancer risk (adjusted hazard ratio [AHR] 1.40; 95% confidence interval [CI], 1.03–1.89) (Tseng et al., 2014). More significantly, metabolic syndrome—characterized by insulin resistance, dyslipidemia, hyperglycemia, and central obesity—serves as an independent prognostic factor, with affected patients demonstrating substantially reduced 5-year OS compared to metabolically healthy individuals (78.4% vs. 85.7%, P = 0.001) (Huang et al., 2021). Among these metabolic abnormalities, lipid dysregulation shows particular clinical significance: studies from Southern China indicate that hypercholesterolemia significantly increases NPC risk, while decreased pretreatment high-density lipoprotein cholesterol levels also predict worse survival outcomes (Liu et al., 2016; Wang C.-T. et al., 2019).

The pathophysiological mechanisms linking metabolic dysfunction to carcinogenesis involve insulin resistance and compensatory hyperinsulinemia (Zhang et al., 2021). Chronic hyperinsulinemia suppresses hepatic synthesis of insulin-like growth factor binding proteins, thereby increasing free Insulin-like Growth Factor-1 (IGF-1) bioavailability, which subsequently activates IGF-1 receptors (IGF-1R) to promote cellular proliferation and metastatic potential (Djiogue et al., 2013). In NPC, elevated IGF-1 levels demonstrate diagnostic value, while increased Insulin-like Growth Factor-Binding Protein (IGFBP-1): IGF-1 ratios independently predict adverse survival (Feng et al., 2017). Mechanistic studies reveal that Epstein-Barr virus (EBV) infection—causally implicated in over 95% of endemic cases—directly induces IGF-1 expression through viral small RNA pathways, establishing autocrine proliferative loops (Iwakiri et al., 2005). This virus-metabolic interaction is further amplified by EBV-mediated alterations in glucose metabolism, where viral latent membrane proteins upregulate glucose transporter expression and enhance glycolytic flux (Zhang et al., 2017).

Although the hyperinsulinemic-euglycemic clamp (HEC) represents the gold standard for assessing peripheral insulin sensitivity, its clinical application is limited by practical constraints. The homeostatic model assessment for insulin resistance (HOMA-IR) similarly faces limitations including high costs and potential interference from exogenous insulin. In contrast, the TyG index, calculated from fasting triglyceride and glucose values, has emerged as a practical surrogate marker with validity comparable to HOMA-IR (Guerrero-Romero et al., 2010; Er et al., 2016). This index uniquely captures the combined effects of lipotoxicity and glucotoxicity, both crucial modulators of insulin resistance, and has demonstrated prognostic value across various malignancies (Fritz et al., 2020).

Despite its recognized utility in multiple clinical settings, no studies have investigated the prognostic significance of the TyG index in NPC populations. This study therefore examined the prognostic value of the TyG index in a large-scale, real-world NPC cohort to elucidate metabolic contributions to NPC prognosis and facilitate development of clinically applicable prognostic tools.

## 2 Methods

### 2.1 Patients

This retrospective study consecutively included patients with NPC who received platinum-based CCRT at the Sun Yat-sen University Cancer Center in China between January 2010 and December 2014. The cohort was defined by several strict inclusion criteria: (1) treatment-naïve non-metastatic NPC, confirmed through both histopathological and radiographic examinations; (2) availability of comprehensive pretreatment clinical and laboratory data; (3) receipt of radical intensity-modulated radiotherapy combined with weekly or triweekly platinum-based concurrent chemotherapy; (4) absence of any subsequent second primary cancers or a history of prior malignant tumors; and (5) the availability of complete and regular follow-up records. All patients were restaged in accordance with the 8th edition of the AJCC/UICC TNM staging system. The study protocol received ethical approval from the Research Ethics Committee of the Sun Yat-sen University Cancer Center, with a waiver of written informed consent. This observational study was reported in compliance with the Strengthening the Reporting of Observational Studies in Epidemiology (STROBE) guidelines.

### 2.2 Data collection

Clinical and demographic information was meticulously extracted from the medical records of all eligible patients. The primary laboratory data, including fasting triglyceride and blood glucose levels, were collected from pretreatment biochemical tests. Plasma Epstein-Barr virus DNA (EBV-DNA) levels were measured using a real-time quantitative polymerase chain reaction method.

The TyG index was mathematically determined using the following formula (Simental-Mendía et al., 2008): TyG index = ln [triglycerides (mg/dL) × blood glucose (mg/dL)/2].

Survival outcomes were defined as follows: OS was the period from the date of initial diagnosis to the date of death from any cause or the date of the last follow-up. LRFS was defined as the time from the date of diagnosis to the first documented occurrence of a locoregional relapse. DMFS was the time from the date of diagnosis to the first documented occurrence of distant metastasis. For both LRFS and DMFS, patients who did not experience an event were censored at the time of their last follow-up.

### 2.3 Statistical analysis

Continuous variables were summarized using medians and interquartile ranges (IQR), while categorical variables were presented as frequencies and percentages. The optimal cutoff value for the TyG index was determined using the maximally selected rank statistics with survival status as the primary endpoint, employing the “maxstat” package (Hothorn and Zeileis, 2008; Hua et al., 2021). This method was chosen to derive a data-specific threshold that best discriminates between patient outcomes within this particular cohort, rather than relying on an arbitrary or a pre-defined value. Survival curves were generated using the Kaplan-Meier method and compared using log-rank tests.

To assess the relationship between clinicopathological factors and survival, both univariate and multivariate Cox proportional hazards models were utilized. Variables with a P-value <0.20 in the univariate analysis were considered for inclusion in the multivariate model. The proportional hazards assumption for the multivariate model was validated using Schoenfeld residuals. The variance inflation factors (VIFs) were calculated to detect potential multicollinearity among the variables, with no substantial multicollinearity (all VIFs <10) being observed.

Prognostic nomograms were constructed based on the significant independent prognostic factors identified in the multivariate analyses. The discriminative ability of the models was quantified using Harrell’s C-index. The predictive performance of the nomograms was further assessed through calibration curves and Decision Curve Analysis (DCA). A two-tailed P-value <0.05 was considered to indicate statistical significance. All statistical computations were performed using R statistical software version 4.2.1.

## 3 Results

### 3.1 Patient characteristics

A total of 833 non-metastatic NPC patients who were treated with platinum-based CCRT were enrolled in this retrospective study. The baseline clinicopathological characteristics of the patient cohort are summarized in Table 1. The median age was 45.4 years, with 411 individuals (49.3%) aged 45 years or older. The cohort was predominantly male, comprising 616 patients (73.9%). The vast majority of patients had World Health Organization (WHO) histological type III pathology (98.4%). Furthermore, 269 patients (32.3%) had pretreatment plasma EBV-DNA levels of 4,000 copies/mL or greater.

**TABLE 1:** Patient demographics and clinical characteristics.

Characteristic	No. of Patients (%)
Age	
≥45 years	411 (49.3%)
<45 years	422 (50.7%)
Gender	
Male	616 (73.9%)
Female	217 (26.1%)
Histological type	
WHO Ⅰ/II	13 (1.56%)
WHO Ⅲ	820 (98.4%)
HGB	
<113 g/L	26 (3.12%)
113–151 g/L	535 (64.2%)
≥151 g/L	272 (32.7%)
LDH	
≥245 U/L	50 (6.0%)
<245 U/L	783 (94.0%)
ALB	
≥40 g/L	764 (91.8%)
<40 g/L	68 (8.2%)
T stage	
T1	41 (4.9%)
T2	160 (19.2%)
T3	507 (60.9%)
T4	125 (15.0%)
N stage	
N0	77 (9.2%)
N1	447 (53.7%)
N2	265 (31.8%)
N3	44 (5.3%)
EBV-DNA	
<4,000 copies/mL	564 (67.7%)
≥4,000 copies/mL	269 (32.3%)
TyG index	
>8.92	250 (30.0%)
≤8.92	583 (70.0%)

Abbreviations: WHO, world health organization; HGB, hemoglobin; LDH, serum lactate dehydrogenase levels; EBV-DNA, Epstein-Barr virus DNA; TyG index, triglyceride-glucose index.

The optimal cutoff value for the TyG index was determined to be 8.92 using the maximally selected rank statistics (Hothorn and Zeileis, 2008). This data-driven threshold, which was specifically calibrated to provide the best separation in survival outcomes for this cohort, was then used to dichotomize the patient population into two groups: a high-TyG group (>8.92, n = 250, 30.0%) and a low-TyG group (≤8.92, n = 583, 70.0%) (Supplementary Figure S1).

### 3.2 Prognostic value of TyG index in NPC

The median follow-up period for the entire cohort was 63.1 months (IQR: 49.3–72.3 months). During this time, the median OS was 63.0 months, with 78 (9.4%) deaths occurring across the entire cohort. There were 78 (9.4%) locoregional recurrences and 92 (11.0%) distant metastases.

A clear and significant difference in survival was observed between the two TyG index groups. The high-TyG group experienced a higher rate of adverse events, with 36 deaths (14.4%), 38 locoregional recurrences (15.2%), and 37 distant metastases (14.8%). In contrast, the low-TyG group experienced 42 deaths (7.2%), 40 locoregional recurrences (6.9%), and 55 distant metastases (9.4%), representing significantly fewer events.

**FIGURE 1:**
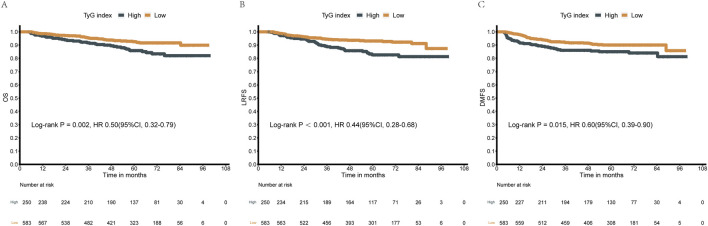
Kaplan-Meier survival curves comparing high and low TyG index groups. **(A)** Overall Survival (OS) analysis (log-rank P = 0.002, HR = 0.50). **(B)** Local Recurrence-Free Survival (LRFS) analysis (log-rank P < 0.001, HR = 0.44). **(C)** Distant Metastasis-Free Survival (DMFS) analysis (log-rank P = 0.015, HR = 0.60). The TyG index significantly stratified all three survival outcomes. The horizontal axis represents follow-up time in months, and the vertical axis indicates the estimated survival probability. Numbers of patients at risk are displayed below the respective survival curves.

Kaplan-Meier survival analysis demonstrated that patients in the low-TyG group had consistently superior survival outcomes compared to those in the high-TyG group for all three endpoints (Figure 1). The TyG index was significantly associated with OS (HR = 0.50; 95% CI: 0.32–0.79; P = 0.002), LRFS (HR = 0.44; 95% CI: 0.28–0.68; P < 0.001), and DMFS (HR = 0.60; 95% CI: 0.39–0.90; P = 0.015).

### 3.3 Cox regression analyses of OS, LRFS and DMFS in NPC

Univariate and multivariate Cox regression analyses were conducted to evaluate the independent prognostic significance of the TyG index and other clinicopathological factors. The assumption of proportional hazards was met for the multivariate models.

The results of the multivariate analysis for OS revealed that the TyG index was an independent prognostic factor (HR = 0.534; 95% CI: 0.339–0.841; P = 0.007). Other factors independently associated with OS included N stage, with N2 (HR = 3.066; P = 0.037) and N3 (HR = 4.665; P = 0.013) stages carrying a significantly higher risk compared to N0 stage. T stage T4 (HR = 7.830; P = 0.046) was also an independent predictor for OS (Table 2).

**TABLE 2:** Univariate and multivariate cox regression analyses of overall survival.

Characteristic	Univariate analysis		Multivariate analysis	
	Hazard ratio (95% CI)	*P* value	Hazard ratio (95% CI)	*P* value
Age				
≥45 years	1		1	
<45 years	0.589 (0.374–0.926)	0.022	0.645 (0.405–1.027)	0.064
Gender				
Male	1			
Female	0.727 (0.420–1.260)	0.256		
Histological type				
WHO Ⅰ/II	1		1	
WHO Ⅲ	2.479 (0.781–7.868)	0.123	2.250 (0.694–7.289)	0.176
HGB				
<113 g/L	1			
113–151 g/L	2.196 (0.303–15.940)	0.437		
≥151 g/L	3.184 (0.436–23.280)	0.254		
LDH				
≥245 U/L	1			
<245 U/L	0.842 (0.340–2.083)	0.710		
ALB				
≥40 g/L	1			
<40 g/L	1.065 (0.463–2.450)	0.882		
T stage				
T1	1		1	
T2	2.639 (0.338–20.610)	0.355	2.735 (0.348–21.530)	0.339
T3	3.995 (0.551–28.950)	0.170	3.991 (0.548–29.083)	0.172
T4	7.112 (0.952–53.130)	0.056	7.830 (1.040–58.956)	0.046
N stage				
N0	1		1	
N1	1.382 (0.488–3.917)	0.542	1.532 (0.536–4.381)	0.426
N2	2.681 (0.951–7.555)	0.062	3.066 (1.069–8.794)	0.037
N3	4.386 (1.350–14.246)	0.014	4.665 (1.392–15.638)	0.013
EBV-DNA				
<4,000 copies/mL	1		1	
≥4,000 copies/mL	1.659 (1.058–2.601)	0.027	1.280 (0.801–2.044)	0.302
TyG index				
>8.92	1		1	
≤8.92	0.503 (0.322–0.785)	0.002	0.534 (0.339–0.841)	0.007

Hazard ratios estimated by Cox proportional hazards regression. All statistical tests were two-sided.

Abbreviations: WHO, world health organization; HGB, hemoglobin; LDH, serum lactate dehydrogenase levels; EBV-DNA, Epstein-Barr virus DNA; TyG index, triglyceride-glucose index.

For LRFS, the multivariate analysis confirmed the TyG index as an independent predictor (HR = 0.423; 95% CI: 0.271–0.662; P < 0.001). A similar trend was observed for N stage, where N3 stage (HR = 4.324; P = 0.039) was significantly associated with poorer LRFS (Table 3).

**TABLE 3:** Univariate and multivariate cox regression analyses of locoregional recurrence-free survival.

Characteristic	Univariate analysis		Multivariate analysis	
	Hazard ratio (95% CI)	*P* value	Hazard ratio (95% CI)	*P* value
Age				
≥45 years	1			
<45 years	0.838 (0.536–1.312)	0.440		
Gender				
Male	1			
Female	0.898 (0.535–1.506)	0.683		
Histological type				
WHO Ⅰ/II	1			
WHO Ⅲ	1.194 (0.166–8.584)	0.860		
HGB				
<113 g/L	1			
113–151 g/L	2.313 (0.319–16.770)	0.407		
≥151 g/L	3.089 (0.422–22.630)	0.267		
LDH				
≥245 U/L	1		1	
<245 U/L	0.494 (0.238–1.026)	0.059	0.565 (0.268–1.191)	0.134
ALB				
≥40 g/L	1			
<40 g/L	1.109 (0.448–2.746)	0.823		
T stage				
T1	1		1	
T2	1.587 (0.549–4.589)	0.394	1.708 (0.495–5.893)	0.397
T3	1.809 (0.664–4.933)	0.247	1.454 (0.451–4.691)	0.531
T4	2.450 (0.855–7.021)	0.095	1.955 (0.553–6.917)	0.298
N stage				
N0	1		1	
N1	2.409 (0.745–7.788)	0.142	2.332 (0.716–7.600)	0.160
N2	3.019 (0.918–9.931)	0.069	2.869 (0.859–9.578)	0.087
N3	4.753 (1.228–18.394)	0.024	4.324 (1.080–17.303)	0.039
EBV-DNA				
<4,000 copies/mL	1		1	
≥4,000 copies/mL	1.548 (1.102–2.174)	0.012	1.141 (0.705–1.846)	0.591
TyG index				
>8.92	1		1	
≤8.92	0.439 (0.282–0.684)	<0.001	0.423 (0.271–0.662)	<0.001

Hazard ratios estimated by Cox proportional hazards regression. All statistical tests were two-sided.

Abbreviations: WHO, world health organization; HGB, hemoglobin; LDH, serum lactate dehydrogenase levels; EBV-DNA, Epstein-Barr virus DNA; TyG index, triglyceride-glucose index.

The multivariate analysis for DMFS also identified the TyG index as an independent prognostic factor (HR = 0.575; 95% CI: 0.377–0.877; P = 0.010). N stage N3 (HR = 3.595; P = 0.021) and EBV-DNA levels ≥4,000 copies/mL (HR = 1.873; P = 0.004) were also independent predictors of DMFS (Table 4).

**TABLE 4:** Univariate and multivariate cox regression analyses of distant metastasis-free survival.

Characteristic	Univariate analysis		Multivariate analysis	
	Hazard ratio (95% CI)	*P* value	Hazard ratio (95% CI)	*P* value
Age				
≥45 years	1			
<45 years	0.983 (0.653–1.480)	0.934		
Gender				
Male	1			
Female	0.829 (0.509–1.349)	0.451		
Histological type				
WHO Ⅰ/II	1			
WHO Ⅲ	1.585 (0.504–4.985)	0.431		
HGB				
<113 g/L	1		1	
113–151 g/L	2.654 (0.367–19.200)	0.334	1.997 (0.274–14.537)	0.495
≥151 g/L	3.947 (0.542–28.730)	0.175	2.759 (0.376–20.257)	0.318
LDH				
≥245 U/L	1		1	
<245 U/L	0.583 (0.314–1.082)	0.087	0.836 (0.382–1.828)	0.653
ALB				
≥40 g/L	1			
<40 g/L	1.047 (0.549–1.998)	0.888		
T stage				
T1	1		1	
T2	2.223 (0.671–7.365)	0.191	1.853 (0.544–6.313)	0.324
T3	1.928 (0.607–6.124)	0.266	1.434 (0.445–4.623)	0.546
T4	3.512 (1.070–11.533)	0.038	2.861 (0.850–9.634)	0.090
N stage				
N0	1		1	
N1	1.264 (0.496–3.222)	0.623	1.163 (0.451–3.002)	0.754
N2	2.446 (0.964–6.206)	0.060	2.093 (0.809–5.418)	0.128
N3	4.936 (1.738–14.017)	0.003	3.595 (1.211–10.670)	0.021
EBV-DNA				
<4,000 copies/mL	1		1	
≥4,000 copies/mL	2.013 (1.420–2.855)	<0.001	1.873 (1.216–2.883)	0.004
TyG index				
>8.92	1		1	
≤8.92	0.596 (0.394–0.903)	0.015	0.575 (0.377–0.877)	0.010

Hazard ratios estimated by Cox proportional hazards regression. All statistical tests were two-sided.

Abbreviations: WHO, world health organization; HGB, hemoglobin; LDH, serum lactate dehydrogenase levels; ALB, albumin; EBV-DNA, Epstein-Barr virus DNA; TyG index, triglyceride-glucose index.

### 3.4 Development of novel prognostic models based on TyG index

**FIGURE 2:**
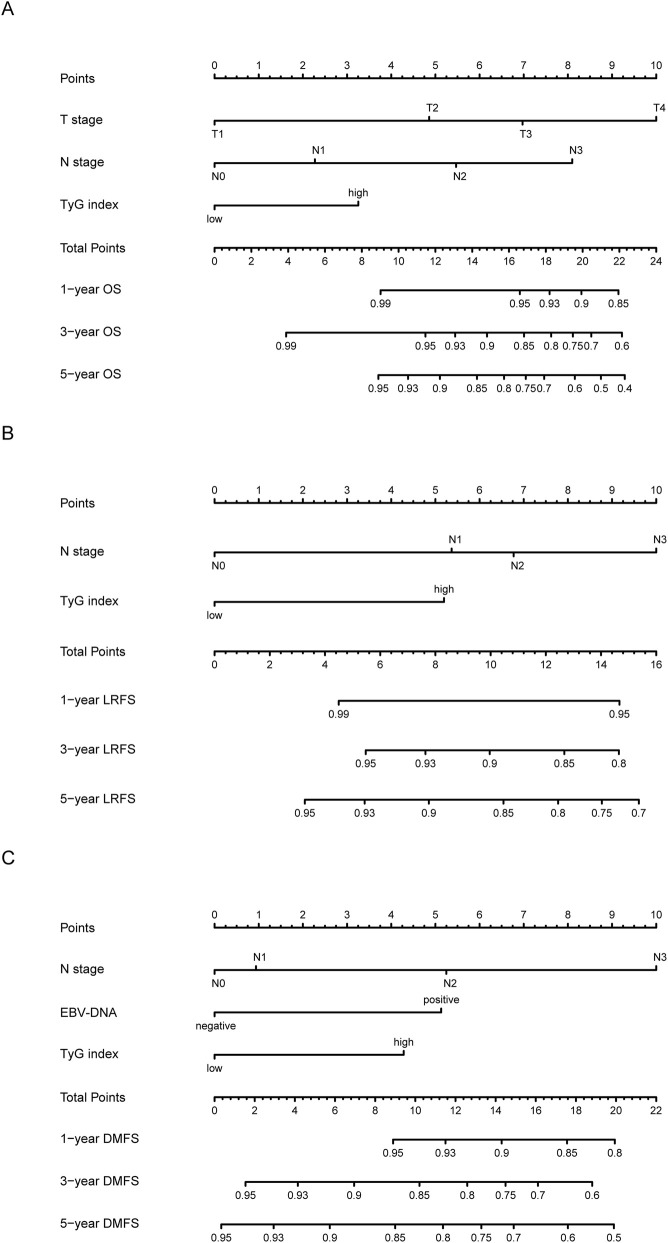
Prognostic nomograms for individualized prediction of 1-, 3-, and 5-year survival outcomes. These nomograms were constructed using multivariable modeling results based on selected clinical factors. **(A)** Nomogram predicting 1-year, 3-year, and 5-year Overall Survival (OS), incorporating T stage, N stage, and the TyG index. **(B)** Nomogram for Local Recurrence-Free Survival (LRFS), incorporating N stage and the TyG index. **(C)** Nomogram for Distant Metastasis-Free Survival (DMFS), uniquely incorporating N stage, EBV-DNA status, and the TyG index. Prediction is calculated by summing points from each predictor variable to achieve a total score, which is then mapped to the final survival probability scale.

Based on the independent prognostic factors identified in the multivariate analyses, new nomograms for the individualized prediction of 1-, 3-, and 5-year survival probabilities were constructed (Figure 2). These models were designed to be simple and clinically accessible, allowing for the calculation of a cumulative prognostic score for each patient by aggregating the scores from each of the predictive variables. This aggregate score could then be used to forecast survival probabilities, providing a straightforward tool for clinicians to estimate a patient’s prognosis.

### 3.5 Assessment of predictive performance of the prognostic models

**FIGURE 3:**
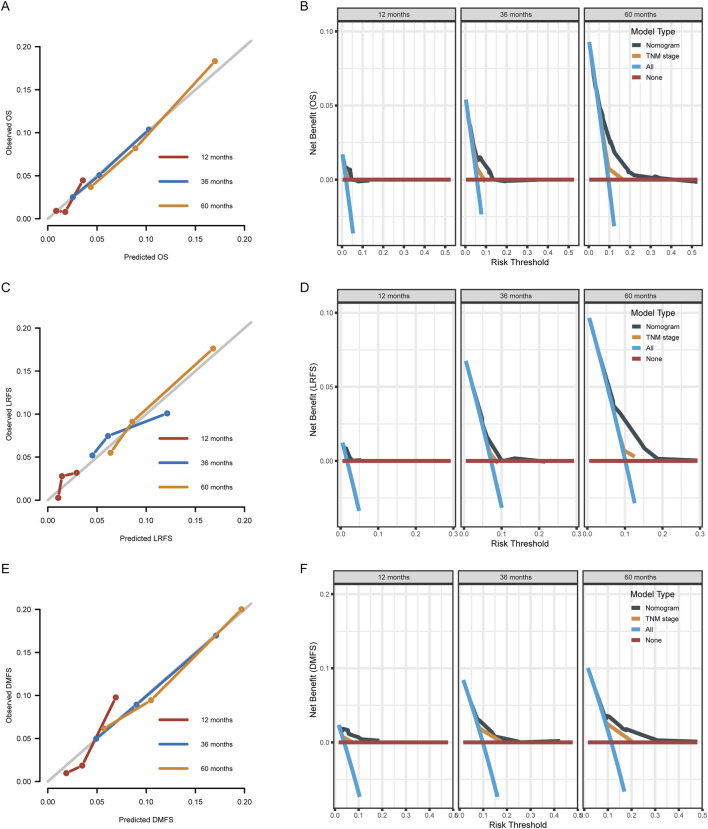
Validation of nomogram models via calibration plots and Decision Curve Analysis (DCA) at 12, 36, and 60 months. **(A,C,E)** Calibration curves for Overall Survival (OS), Locoregional Recurrence-Free Survival (LRFS), and Distant Metastasis-Free Survival (DMFS); proximity to the diagonal line represents ideal prediction accuracy. **(B,D,F)** DCA plots illustrate the clinical Net Benefit of the nomogram. Model performance is compared against the TNM stage and reference strategies (treat all/treat none), quantifying superior clinical utility across decision thresholds.

The developed prognostic models demonstrated a robust discriminatory capacity (Figure 3). The C-indices for predicting survival were 0.722 (95% CI: 0.661–0.783) for OS, 0.655 (95% CI: 0.582–0.728) for LRFS, and 0.679 (95% CI: 0.617–0.742) for DMFS. This performance was quantitatively superior to the conventional TNM staging system, which yielded C-indices of 0.634 (95% CI: 0.526–0.742) for OS, 0.562 (95% CI: 0.453–0.671) for LRFS, and 0.599 (95% CI: 0.513–0.685) for DMFS. The calibration plots further illustrated a strong concordance between the nomogram-predicted and the actual observed survival outcomes at 1, 3, and 5 years. Additionally, DCA curves indicated that the TyG-based models provided a net benefit superior to that of the traditional TNM staging system.

## 4 Discussion

This study represents the first comprehensive analysis evaluating the prognostic utility of the TyG index in a large NPC cohort receiving concurrent chemoradiotherapy. Our findings demonstrate that elevated pretreatment TyG values independently predict reduced overall survival, increased locoregional recurrence, and enhanced distant metastatic risk. This prognostic capacity remained statistically robust after adjusting for established clinical determinants, including TNM staging and pretreatment EBV-DNA levels. The sustained significance of TyG index within multivariate analyses indicates this metabolic marker captures biologically meaningful systemic information distinct from tumor anatomical features, thereby offering complementary risk assessment capabilities that enhance traditional prognostic paradigms.

The mechanistic connections between metabolic dysfunction and cancer outcomes align with accumulating evidence supporting these fundamental pathophysiologic relationships. Metabolic syndrome and insulin resistance show well-documented associations with increased cancer incidence and reduced survival across various malignancy types (Esposito et al., 2012). Our investigation extends this framework by specifically characterizing TyG index prognostic relevance in NPC, an area previously lacking systematic evaluation.

Compensatory hyperinsulinemia from insulin resistance reduces hepatic IGF binding protein production, increasing bioavailable IGF-1 that stimulates IGF-1R signaling pathways promoting cellular growth and metastatic spread (Clayton et al., 2011; Hua et al., 2020). In NPC, elevated serum IGF-1 associates with poor outcomes (M’hamdi et al., 2016; Feng et al., 2017), while EBV infection enhances IGF-1 expression through autocrine mechanisms (Iwakiri et al., 2005; Yuan et al., 2008). Laboratory investigations confirm IGF-1R inhibition reduces proliferation, improves radiosensitivity, and limits metastasis (Wang Z. et al., 2019; Yang et al., 2024), with early clinical trials demonstrating preliminary antitumor efficacy (de Bono et al., 2020).

Beyond IGF pathway alterations, the TyG index simultaneously captures glucotoxicity and lipotoxicity effects. EBV-mediated metabolic reprogramming enhances glycolytic activity, producing lactate that impairs anti-tumor immune responses while generating nicotinamide adenine dinucleotide phosphate (NADPH) to counteract oxidative stress during metastatic processes (Zhang et al., 2017). Persistent hyperglycemia supplies continuous metabolic fuel supporting proliferative activity. Lipid dysfunction facilitates enhanced fatty acid synthesis and oxidation, maintaining energy generation during metabolic stress conditions. Triglyceride-enriched lipoproteins deliver exogenous substrates through upregulated cellular receptors, supporting membrane biosynthesis or undergoing beta-oxidation to produce adenosine triphosphate (ATP) for invasive processes. Disrupted lipid homeostasis additionally provides ferroptosis resistance mechanisms.

The TyG index delivers distinctive clinical value by concurrently assessing both metabolic disturbances, providing integrated systemic dysfunction evaluation. Through incorporating fasting triglyceride and glucose—two routinely available parameters reflecting distinct yet interconnected metabolic abnormality dimensions—the TyG index achieves superior prognostic differentiation compared with individual component assessment. This integrative capability proves especially pertinent in NPC, where viral-induced metabolic alterations affect glycolytic and lipogenic networks, establishing a systemic metabolic environment the TyG index effectively quantifies (Zhang et al., 2017).

The identified prognostic relationships suggest promising therapeutic possibilities. The concept that pharmacologic TyG reduction might improve survival outcomes warrants rigorous prospective investigation. Multiple studies show metformin induces cell cycle arrest occurred at the G1 phase, inhibiting NPC growth, enhancing radiation sensitivity through DNA repair pathway disruption, and reversing cisplatin resistance via platelet endothelial cell adhesion molecule-1 (PECAM-1)-mediated multidrug resistance protein reduction (Zhao et al., 2011; Li et al., 2014; Sun et al., 2020). Similarly, statins demonstrate significant growth-inhibitory properties and enhance cisplatin effectiveness (Wang et al., 2011; Ma et al., 2019). These findings provide mechanistic rationale that targeting metabolic pathways reflected by TyG index may constitute promising therapeutic approaches for improving outcomes among patients displaying elevated metabolic dysregulation.

Several methodological limitations require consideration. First, the retrospective, single-center design introduces potential selection bias and limits generalizability across diverse populations. External validation through independent multicenter cohorts will prove essential for clinical implementation and enable assessment of model applicability across varied treatment protocols and geographical regions. Second, while numerous NPC prognostic models incorporate complex imaging parameters limiting practical applicability, the TyG index offers distinct advantages: derivation from standard pretreatment laboratory values, reflection of systemic metabolic status complementing tumor anatomy, and seamless integration into existing clinical workflows. Future comparative analyses evaluating TyG-based models versus comprehensive multivariable systems would clarify relative clinical utility. Third, absence of longitudinal TyG monitoring prevented evaluation of whether dynamic metabolic changes during treatment correlate with recurrence risk or therapeutic response. Future prospective studies should incorporate systematic serial TyG measurements at predetermined intervals to characterize temporal patterns and prognostic significance. Additionally, incomplete retrospective documentation of concurrent metabolic medication use precluded reliable assessment of potential confounding influences. Given substantial preclinical evidence suggesting these agents may influence NPC cellular behavior, future prospective investigations should document comprehensive medication histories and examine whether metabolic interventions differentially impact outcomes across TyG strata, potentially identifying high-risk subgroups most likely to benefit from metabolic optimization.

This investigation establishes the TyG index as a robust independent prognostic marker in NPC patients receiving concurrent chemoradiotherapy. The index quantifies systemic metabolic dysregulation affecting tumor biology through multiple interconnected pathways, providing clinically meaningful prognostic information complementary to conventional anatomical staging. Further multicenter, prospective studies are needed to validate these findings and explore potential therapeutic interventions targeting metabolic pathways in this population.

## 5 Conclusion

In summary, the present study demonstrates the considerable prognostic relevance of the triglyceride-glucose index for patients with nasopharyngeal carcinoma undergoing concurrent chemoradiotherapy. The TyG index is an independent and robust prognostic biomarker for overall survival, locoregional recurrence-free survival, and distant metastasis-free survival. The prognostic nomograms developed using the TyG index exhibited superior predictive performance compared to the conventional staging system. This highlights the potential of the TyG index as a valuable, easily accessible, and non-invasive tool for guiding individualized treatment choices and enhancing patient prognosis. Future investigative efforts are warranted to explore in detail how the TyG index may specifically mediate the customization of targeted therapeutic strategies and, consequently, influence the attainment of durable survival benefits in the context of NPC treatment paradigms.

## Data Availability

The raw data supporting the conclusions of this article will be made available by the authors, without undue reservation.

## Supplementary material

SUPPLEMENTARY FIGURE S1 Determination of the cutoff value of TyG index according to maximally selected log-rank statistics.
