# Supplementary figures and images for: Assessing insulin resistance: the triglyceride-glucose index as a predictor of survival in nasopharyngeal carcinoma
Source: Front Physiol. 2026 Jan 5;16:1716333. doi: 10.3389/fphys.2025.1716333 (PMC12812891; doi:10.3389/fphys.2025.1716333)

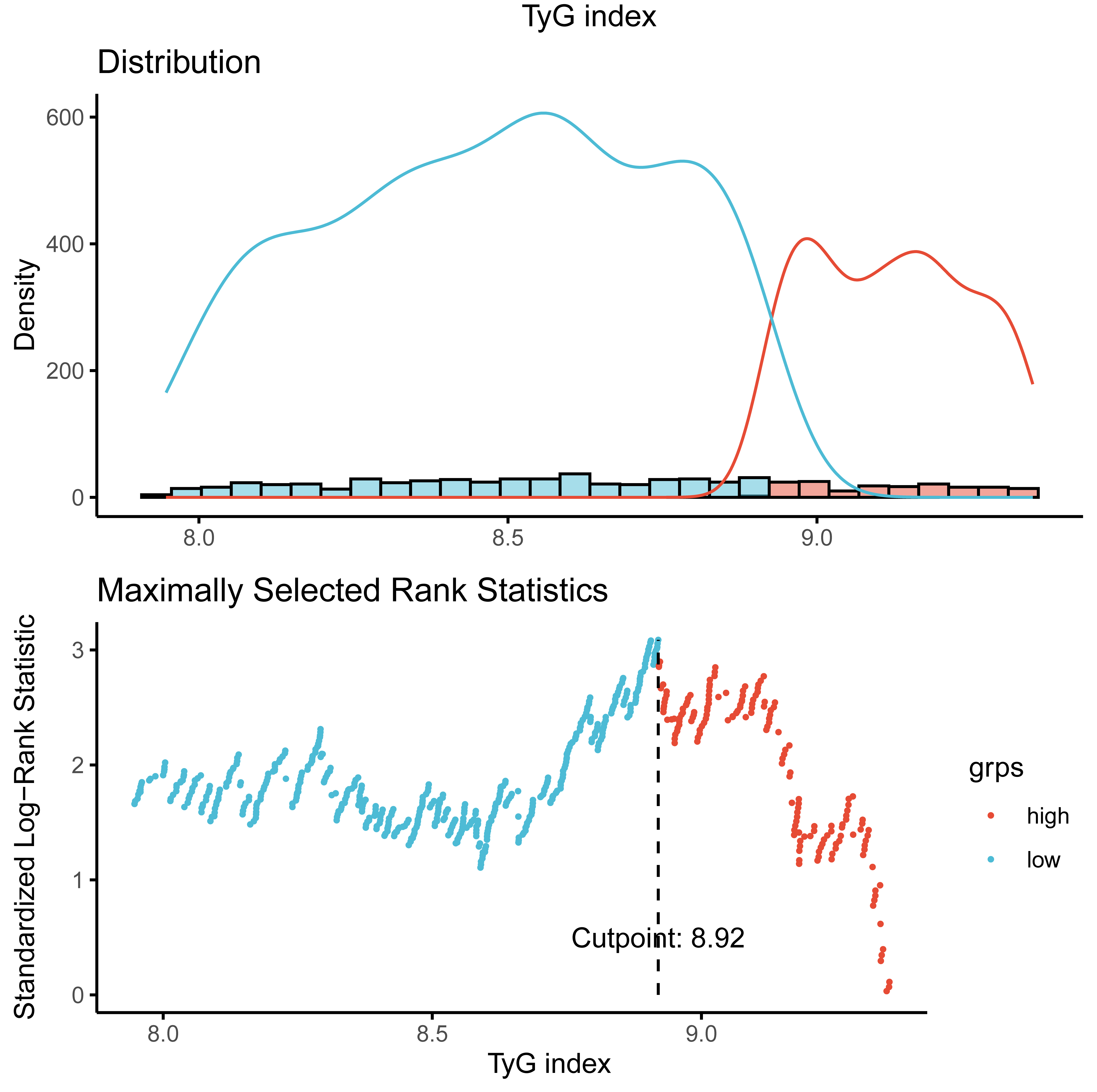

Supplement: Supplementary file 1 [file Image1.tif]
